# Preliminary Evaluation of the Scandinavian Guidelines for Initial Management of Minimal, Mild, and Moderate Head Injuries with Glial Fibrillary Acidic Protein

**DOI:** 10.1089/neur.2023.0077

**Published:** 2024-01-16

**Authors:** Mira Keski-Pukkila, Justin E. Karr, Jussi P. Posti, Ksenia Berghem, Anna-Kerttu Kotilainen, Kaj Blennow, Henrik Zetterberg, Grant L. Iverson, Teemu M. Luoto

**Affiliations:** ^1^Faculty of Medicine and Health Technology, Tampere University and Tampere University Hospital, Tampere, Finland.; ^2^ Department of Psychology, University of Kentucky, Lexington, Kentucky, USA.; ^3^ Neurocenter, Department of Neurosurgery, and Turku Brain Injury Center, Turku University Hospital, and University of Turku, Turku, Finland.; ^4^ Medical Imaging Centre, Department of Radiology, Tampere University Hospital, Tampere, Finland.; ^5^ Institute of Neuroscience and Physiology, Department of Psychiatry and Neurochemistry, the Sahlgrenska Academy at the University of Gothenburg, Mölndal, Sweden.; ^6^ Clinical Neurochemistry Laboratory, Sahlgrenska University Hospital, Mölndal, Sweden.; ^7^ UK Dementia Research Institute, Institute of Neurology, University College London, London, United Kingdom.; ^8^ Department of Molecular Neuroscience, Queen Square Institute of Neurology, University College London, London, United Kingdom.; ^9^ Hong Kong Center for Neurodegenerative Diseases, Clear Water Bay, Hong Kong, China.; ^10^ Wisconsin Alzheimer's Disease Research Center, University of Wisconsin School of Medicine and Public Health, University of Wisconsin–Madison, Madison, Wisconsin, USA.; ^11^ Department of Physical Medicine and Rehabilitation, Harvard Medical School, Boston, Massachusetts, USA.; ^12^ Spaulding Rehabilitation Hospital and the Schoen Adams Research Institute at Spaulding Rehabilitation, Charlestown, Massachusetts, USA.; ^13^ Home Base, A Red Sox Foundation and Massachusetts General Hospital Program, Charlestown, Massachusetts, USA.; ^14^ Department of Neurosurgery, Tampere University Hospital and Tampere University, Tampere, Finland.

**Keywords:** computed tomography, emergency treatment, glial fibrillary acidic protein, guideline, traumatic brain injury

## Abstract

Glial fibrillary acidic protein (GFAP) has become the most promising biomarker for detecting traumatic abnormalities on head computed tomography (CT) in patients with traumatic brain injury (TBI), but most studies have not addressed the potential added value of combining the biomarker with clinical variables that confer risk for intracranial injuries. The Scandinavian Guidelines for Initial Management of Minimal, Mild, and Moderate Head Injuries in Adults were the first clinical decision rules in the field with an incorporated biomarker, the S100 astroglial calcium-binding protein B (S100B), which is used in the Mild (Low Risk) group defined by the guidelines. Our aim was to evaluate the performance of the guidelines when S100B was substituted with GFAP. The sample (*N* = 296) was recruited from the Tampere University Hospital's emergency department between November 2015 and November 2016, and there were 49 patients with available GFAP results who were stratified in the Mild (Low Risk) group (thus patients undergoing biomarker triaging). A previously reported cutoff of plasma GFAP ≥140 pg/mL was used. Within the Mild (Low Risk) group (*n* = 49), GFAP sensitivity (with 95% confidence intervals in parentheses) for detecting traumatic CT abnormalities was 1.0 (0.40–1.00), specificity 0.34 (0.19–0.53), the negative predictive value (NPV) 1.0 (0.68–1.00), and the positive predictive value (PPV) 0.16 (0.05–0.37). The sensitivity and specificity of the modified guidelines with GFAP, when applied to all imaged patients (*n* = 197) in the whole sample, were 0.94 (0.77–0.99) and 0.20 (0.15–0.28), respectively. NPV was 0.94 (0.80–0.99) and PPV 0.18 (0.13–0.25). In the Mild (Low Risk) group, none of the patients with GFAP results below 140 pg/mL had traumatic abnormalities on their head CT. These findings were derived from a small patient subgroup. Future researchers should replicate these findings in larger samples and assess whether GFAP has added or comparable value to S100B in acute TBI management.

## Introduction

Traumatic brain injury (TBI) is among the most common reasons for seeking emergency department (ED) care and a leading cause of morbidity globally.^1,2^ Head computed tomography (CT) is the imaging modality of choice for identifying patients with acute traumatic intracranial pathology in the ED.^3,4^ However, there are reasons to be judicious in referrals for head CT, such as reducing exposure to radiation^5^ and reducing healthcare costs.^6^ Researchers are exploring blood-based biomarkers as an alternative approach to diagnose intracranial traumatic lesions. These efforts aim to discover and validate the most promising blood-based biomarker for detecting acute intracranial traumatic lesions in patients with TBI.^7,8^

The S100 astroglial calcium-binding protein B (S100B) was the first blood biomarker to be combined into the risk stratification for triaging patients with TBIs for referral for head CT in the 2013 Scandinavian Guidelines for Initial Management of Minimal, Mild, and Moderate Head Injuries in Adults^9^ (i.e., Scandinavian Guidelines). However, blood S100B levels are affected by extracranial injuries and cannot be used reliably in patients with polytrauma, and because of a half-life of 2–6 h, its measurement must be done within 6 h from injury.^10–12^

Glial fibrillary acidic protein (GFAP) has emerged as the leading blood biomarker candidate for TBI,^13^ it has been included in the U.S. Food and Drug Administration (FDA)-approved biomarker kit for identifying traumatic CT abnormalities in patients with mild traumatic brain injury (mTBI) in 2018,^14–16^ and it was also included in a FDA-cleared rapid test for the same use.^17^ Per a meta-analysis of nine studies on GFAP prediction of CT abnormalities, a threshold of 22 pg/mL maximized sensitivity at 93% with a specificity of 36%.^8^ However, the differences in assay choices and sample types (i.e., serum or plasma) between studies make it difficult to assess the threshold-performance relationship of GFAP.^8^ Although studies have shown GFAP to outperform S100B when studied as a sole predictor for traumatic CT abnormalities,^18,19^ GFAP has recently been incorporated into clinical guidelines, the French Recommendations for the management of patients sustaining mTBI by the French Society of Emergency Medicine and the French Society of Anaesthesiology and Critical Care Medicine.^20^ GFAP is detectable within 1 h post-TBI and peaks within 20–24 h with a half-life of 24–48 h.^21^ A clinically reliable sampling time for GFAP has not been determined, but a longer half-life allows it to be determined in a wider time window than S100B, theoretically making it more useful in various clinical situations.

The Scandinavian Guidelines are designed to guide the initial management of minimal, mild, and moderate head and brain injuries. After the initial clinical evaluation, a decision is made about whether to send the patient for head CT. According to the Scandinavian Guidelines, injuries that are Moderate, Mild (High Risk), and Mild (Medium Risk) are referred for CT. Injuries that are deemed Mild (Low Risk) are triaged by the examination of S100B levels (if the injury is within 6 h), and if S100B is elevated, they are sent for head CT. In this study, we evaluated GFAP instead of S100B and extended the sampling time to 24 h in the Mild (Low Risk) group as defined by the 2013 Scandinavian Guidelines for the emergency management of TBIs.^9^ This study relied on data collected from a prospective cohort study designed to validate the Scandinavian Guidelines.^22^ We hypothesized that the modified guidelines with GFAP would have high sensitivity and low-medium specificity in detecting acute intracranial abnormalities. We examined whether the modified guidelines, with GFAP, could safely reduce the frequency of CT scanning of mTBI patients without acute intracranial abnormalities.

## Methods

### Participants

The study sample was recruited from the Tampere University Hospital ED (Tampere, Finland) between November 2015 and November 2016. All consecutive adults (≥18 years) with acute (≤24 h) TBI were eligible for inclusion. Minimum criteria for TBI were determined as either blunt injury to the head or acceleration/deceleration-type injury resulting in a witnessed loss of consciousness, disorientation, or amnesia and a Glasgow Coma Scale (GCS) score of 13–15 as assessed 30 min after injury.^23^ During the study period, 3067 adult patients with TBIs (mean age = 56.9 years, standard deviation [SD] = 23.2, median = 58.0, range = 18–103; women = 46.8%) were treated in the ED and 325 (10.6%) consented to participate in the study. After excluding patients with ED admission >24 h post-injury, a total of 296 patients (mean age = 61.1 years, SD = 22.7, median = 67.0, range = 18–100; women** =** 49.0%) were enrolled in a prospective study designed to validate the Scandinavian Guidelines.^22^ Injury mechanism, post-injury signs and symptoms, and the findings of the physical examination performed in the ED were described by the on-call physician in detailed case reports of each patient. All enrolled patients provided informed written consent according to the Declaration of Helsinki. The study was approved by the Ethics Committee of Pirkanmaa Hospital District, Tampere, Finland (ethical code: R15045).

### Outcomes

An acute traumatic lesion on head CT was determined to be the primary outcome. Secondary outcomes were delayed complications resulting from the head or brain injury, including ED or hospital readmission, repeat head CT, or death within a week after injury. At the 1-week follow-up, a study nurse identified possible complications by a phone call and medical record review.

### Head computed tomography imaging

Referral to a head CT was based on an evaluation by the on-call physician and applying the Scandinavian Guidelines.^9^ Non-contrast head CT was performed with a 64-row CT scanner (Lightspeed VCT; GE, Waukesha, WI). The findings were systematically coded by a neuroradiologist (K.B.) based on the National Institute of Neurological Disorders and Stroke Common Data Elements.^24^

### Biomarker analyses

Venous blood samples were originally drawn from all enrolled patients, and a part of the blood was analyzed at Tampere University Hospital (Tampere, Finland) for a previous study.^22^ The remaining serum and plasma samples were immediately frozen at −70°C for future use. Samples were sent to the Sahlgrenska University Hospital, Mölndal, Sweden (transfer in 20 kg of dry ice by a courier) for further analyses. On September 14–15, 2019, the samples were analyzed using the GFAP Discovery Kit (Quanterix, Billerica, MA) on an HD-1 Simoa instrument to determine plasma GFAP levels. The lower limit of detection and lower limit of quantification were 0.211 and 0.686 pg/mL, respectively. The laboratory technicians performing the analyses were blinded to the clinical data. The details of our GFAP analytics are described more comprehensively in a previous publication and in the Supplementary Material.^25^

### The Mild (Low Risk) group

According to the Scandinavian Guidelines, patients are divided into severity classifications for triaging as follows: 1) Minimal; 2) Mild (Low Risk); 3) Mild (Medium Risk); 4) Mild (High Risk); and 5) Moderate (see Supplementary Fig. S1). S100B levels are used to determine the need for head CT in the Mild (Low risk) group, which is comprised of either 1) patients with GCS of 15 who have suspected/confirmed loss of consciousness, repeated vomiting, or both or 2) patients with GCS of 14. Patients who are ≥65 years of age and who are on antiplatelet medication cannot be classified as Low Risk; they are classified as Medium Risk and are sent for head CT. Patients cannot be in the Low Risk classification if they present with post-traumatic seizures, focal neurological deficits, clinical signs of depressed or basal skull fracture, shunt-treated hydrocephalus, therapeutic anticoagulation, or a coagulation disorder (i.e., patients with any of those features are referred for head CT).^9^ In this study, we examined the performance of plasma GFAP in the Mild (Low Risk) group using a cut-off level of 140 pg/mL. There are no validated cut-off scores for the research-use assays used in this study. This cut-off level was previously derived from another study cohort that was analyzed for GFAP in the same research laboratory using the Human Neurology 4-Plex A assay on a Simoa HD-1.^26^ We opted to use plasma GFAP levels in this study instead of serum because of the lack of previously derived cutoffs with a similar biomarker assay for serum GFAP. The time limit for the interval between injury and blood sampling was moved from the guideline's original 6-h limit to 24 h for the purposes of this study because of the slower release of GFAP after TBI.^21^

### Statistical analyses

Statistical analyses were performed with the Statistical Package for Social Sciences software program (IBM SPSS Statistics for Windows, Versions 22.0-25.0; IBM Corp., Armonk, NY). Distributions of sample characteristics and the plasma GFAP (P-GFAP) in the Mild (Low Risk) group were examined using histograms and Shapiro-Wilk tests of normality, and nearly all distributions were non-normal. Descriptive statistics (frequency [*n*], percentage, median, interquartile range, and range) were used to describe the sample characteristics. Bivariate associations between the biomarkers were calculated using Spearman *rho* correlation coefficients. A *p* < 0.05 was indicative of a statistically significant finding.

Sensitivities were calculated by dividing the number of patients with a head CT indication (indicated by either the P-GFAP result in the Mild [Low Risk] group or the modified guidelines as presented in Supplementary Fig. S1) and a positive head CT result by the total number of positive head CT results, and the specificities by dividing the number of patients without a head CT indication and a negative head CT result by the total number of negative head CT results.

Positive predictive values (PPVs) were calculated by dividing the number of patients with a head CT indication and a positive head CT result by the total number of patients with a head CT indication, and the negative predictive values (NPVs) by dividing the number of patients without a head CT indication and a negative head CT result by the total number of patients without a head CT indication. Confidence intervals (CIs) were calculated by Clinical Calculator 1 of the VassarStats website,^27^ using the continuity-corrected Newcombe-Wilson score method.^28^

## Results

**FIG. 1.**
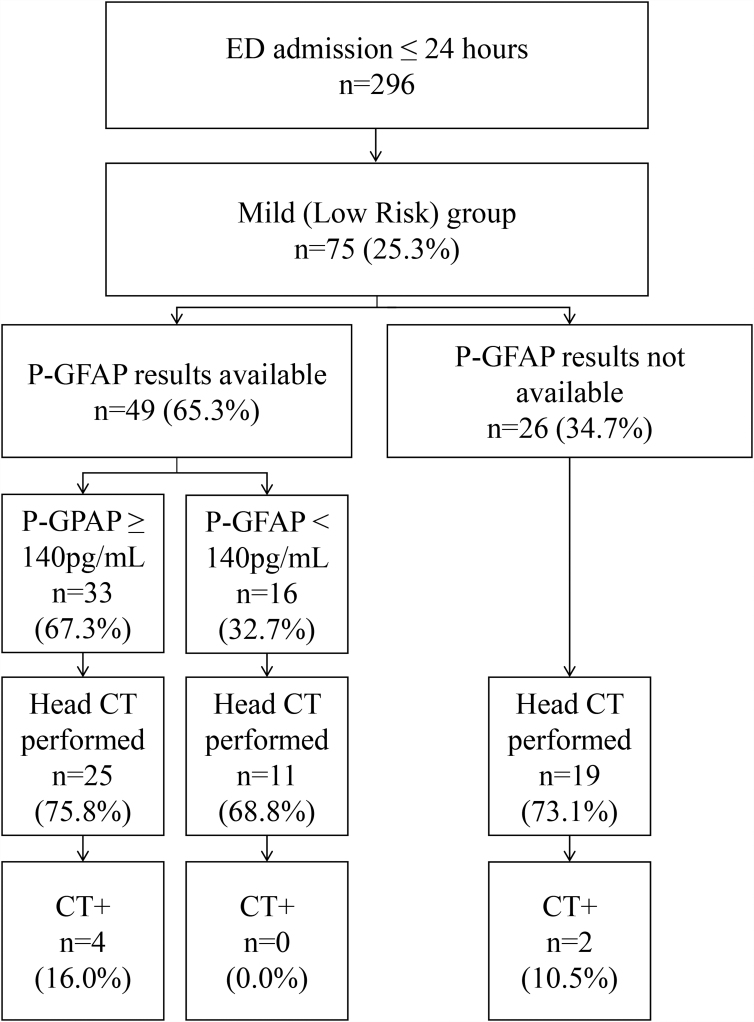
Sample selection. Because of the pragmatic study design, plasma GFAP was unavailable for 26 subjects, because storage samples were not always collected despite being required by the study protocol. ED, emergency department; P-GFAP, plasma glial fibrillary acidic protein; CT, computed tomography.

Patients (*N* = 296) were divided into subgroups according to the Scandinavian Guidelines (see Supplementary Fig. S1). Sample characteristics of the total sample (*N* = 296) are presented in a previous study.^22^ Of the 296, 197 underwent head CT (see row 3 of Supplementary Fig. S1). Sample characteristics for the total sample of 197 patients who underwent head CT are provided in Table 1. Figure 1 presents the sample selection for this study, and the characteristics of the patients with available GFAP results in the Mild (Low Risk) group (*n* = 49) are presented in Table 1. Of these 49 patients, 36 (73.5%) underwent head CT scanning and 4 (11.1%) had acute CT abnormalities. The most common CT abnormality was traumatic subarachnoid hemorrhage (8.3%; *n* = 3).

**Table 1.** Study Sample Characteristics for the Mild (Low Risk) Group (*n* = 49) and the Patients With Available CT Image Results (*n* = 197) in the Total Sample

	Mild (Low Risk) (*n* = 49)		Underwent head CT (*n* = 197)	
Variable	Mdn	IQR	Mdn	IQR
Age (years)	48	25.0–67.5	67	51.5–83.5
Time between injury and blood sampling (h)	3.3	2.0–4.6	3.2	1.8–5.3
Time between injury and ED admission (h)	1.4	0.8–2.5	1.3	0.8–2.8
Time between injury and head CT (h)	4.3	3.0–5.8	4.5	2.5–6.8
Time between injury and ED discharge (h)	6.5	4.7–9.3	7.3	5.0–11.5
Time of ED stay (h)	5.0	3.5–6.3	5.2	3.6–7.0
GCS score	15	range: 14–15	15	range: 8–15
Plasma GFAP concentration (pg/mL)	201.7	105.0–555.5	334.6	156.2–630.9

	*n*	%	*n*	%
Men	29	59.2	92	46.7
Women	20	40.8	105	53.3
Health problems documented in medical records				
Diseases of the circulatory system (I00-99)	19	38.8	138	70.1
Diseases of the respiratory system (J00-99)	8	16.3	40	20.3
Mental and behavioral disorders (F01-99)	21	42.9	88	44.7
Diseases of the nervous system (G00-99)	10	20.4	69	35.0
Endocrine, nutritional, and metabolic diseases (E00-90)	9	18.4	92	46.7
Diseases of the digestive system (K00-93)	4	8.2	46	23.4
Diseases of the genitourinary system (N00-99)	10	20.4	50	25.4
Diseases of the musculoskeletal system and connective tissue (M00-99)	14	28.6	76	38.6
Neoplasms (C00-D48)	8	16.3	60	30.5
Diseases of the blood and blood-forming organs and certain disorders involving the immune mechanism (D50-89)	1	2.0	15	7.6
Diseases of the eye and adnexa (H00-59)	4	8.2	62	31.5
Diseases of the ear and mastoid process (H60-95)	3	6.1	38	19.3
Diseases of the skin and subcutaneous tissue (L00-99)	3	6.1	19	9.6
Congenital malformations, deformations, and chromosomal abnormalities (Q00-99)	1	2.0	7	3.6
Injury, poisoning, and certain other consequences of external causes (S00-T98)	47	95.9	195	99.0
Past cranial neurosurgery	0	0	0	0
Injury mechanism				
Car accident	3	6.1	3	1.5
Ground-level fall	24	49.0	144	73.1
Motorcycle accident	1	2.0	1	0.5
Bicycle accident	2	4.1	5	2.5
Fall from a distance	7	14.3	22	11.2
Sports	6	12.2	6	3.0
Violence	5	10.2	8	4.1
Unknown	1	2.0	6	3.0
Anticoagulant medication	0	0	63	32.0
Antiplatelet medication	1	2.0	41	20.8
Coagulopathy	0	0	3	1.5
Intracranial shunt	0	0	0	0
Loss of consciousness, witnessed or suspected	44	89.8	91	46.2
Post-traumatic amnesia	30	61.2	85	43.1
Focal neurological deficit	0	0	18	9.1
Clinical signs of a skull fracture	0	0	6	3.0
Post-traumatic seizure	0	0	3	1.5
External injury above the clavicle level	35	71.4	156	79.2
Vomiting ≥2 times	4	8.2	9	4.6
Headache	24	49.0	91	46.2
Alcohol intoxication	19	38.8	58	29.4
Neurosurgery because of acute TBI	0	0	3	1.5
Acute extracranial surgery	0	0	3	1.5
Place of follow-up treatment after the ED visit				
Home	36	73.5	122	61.9
University hospital ward	2	4.1	12	6.1
Non-university hospital ward	4	8.2	30	15.2
Health center	4	8.2	23	11.7
Detoxification center	3	6.1	6	3.0
Police station	0	0	1	0.5
Death	0	0	0	0

CT, computed tomography; ED, emergency department; GFAP, glial fibrillary acidic protein; GCS, Glasgow Coma Scale; IQR, interquartile range; Mdn, median; TBI, traumatic brain injury.

**FIG. 2.**
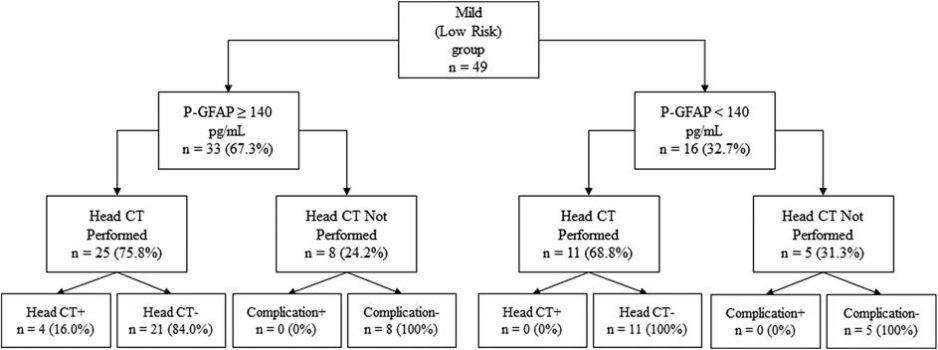
Outcomes of applying P-GFAP in the Scandinavian Guideline for the Mild (Low Risk) group. CT+/– = presence or absence of acute traumatic lesion on head computed tomography (CT). Complication+/– = presence or absence of hospital or ED readmission, repeat head CT, or death within 1 week after injury. P-GFAP, plasma glial acidic fibrillary protein.

Figure 2 presents the outcomes of using P-GFAP in the Scandinavian Guideline for the Mild (Low Risk) group (*n* = 49). Within the group, P-GFAP levels were elevated in 33 of 49 patients (67.3%). Among those patients, 25 (75.8%) underwent CT imaging. Among the 16 patients (32.7%) with P-GFAP levels <140 pg/mL, 11 (68.8%) still underwent CT imaging, and none of the scans showed traumatic abnormalities.

For the 26 (34.7%) patients in the Mild (Low Risk) group who did not have P-GFAP data, a head CT was indicated because the guidelines recommend imaging patients in the Mild (Low Risk) group if biomarker results are not attainable. Of those patients, 19 (73.1%) were imaged and two had traumatic abnormalities on their head CT. Characteristics of patients with traumatic CT abnormalities in the Mild (Low Risk) group are presented in Table 2.

**Table 2.** Characteristics of the Mild (Low Risk) Group Patients With Traumatic Abnormalities on Head CT

Sex	Age	Injury mechanism	GCS	Signs and symptoms at the ED	Time from injury to blood sampling (h)	GFAP (pg/mL)	CT findings	Place of follow-up treatment
Woman	42	Fall	14	LOC, PTA	2.1	3,768	SAH	University hospital ward
Man	61	GLF	15	Suspected LOC, PTA, external injuries above clavicle	5.1	1,490	Acute SDH, SAH	University hospital ward
Man	72	GLF	15	Repeated vomiting, PTA, headache, external injuries above clavicle	3.8	4,296	SAH	Health center
Man	72	Fall	15	Suspected LOC, PTA, external injuries above clavicle	4.3	437	TAI	Home
Man	54	GLF	15	LOC, PTA, external injuries above clavicle	—	—	Skull fracture	Home
Woman	75	GLF	15	Repeated vomiting, headache, external injuries above clavicle	—	—	Skull fracture	University hospital ward

Blood was not stored for two of the patients.

GCS, Glasgow Coma Scale; ED, emergency department; GFAP, plasma glial fibrillary acidic protein; CT, computed tomography; GLF, ground-level fall; LOC, loss of consciousness; PTA, post-traumatic amnesia; SAH, subarachnoid hemorrhage; SDH, subdural hematoma; TAI, traumatic axonal injury.

Within the Mild (Low Risk) group with available P-GFAP data and CT imaging (*n* = 36), the sensitivity and specificity of P-GFAP for detecting traumatic CT abnormalities (with 95% CIs in parentheses) were 1.0 (0.40–1.00) and 0.34 (0.19–0.53), respectively. The NPV was 1.0 (0.68–1.00), and the PPV was 0.16 (0.05–0.37; see Supplementary Table S2).

Within the guideline-specified Minimal TBI group, there were 64 patients with available P-GFAP results. The biomarker results and brain imaging results of these patients are presented in the online Supplementary Table S5. When these patients were combined with those in the Mild (Low Risk) group, there were a total of 55 patients with available P-GFAP and brain imaging results. Within this combined group (*n* = 55), the sensitivity of P-GFAP in detecting intracranial CT abnormalities would have been 1.0 (95% CI, 0.52–1.00), specificity 0.39 (95% CI, 0.26–0.54), the NPV 1.0 (95% CI, 0.79–1.00), and the PPV 0.17 (95% CI, 0.07–0.33), as presented in Supplementary Table S6.

In the 197 patients who underwent imaging, there were 2 patients with traumatic CT abnormalities who did not have a guideline-based indication to undergo a head CT but who underwent a head CT because the physician ordered one. These patients were in the minimal TBI group. Thus, according to the guidelines, they would not need blood sampling or a head CT. Both, however, underwent blood sampling and both patients exceeded the cutoff used in this study (P-GFAP, 886 and 2860 pg/mL). The modified guidelines' sensitivity for detecting traumatic CT abnormalities, calculated within the 197 imaged patients in the whole sample, was 0.94 (0.77–0.99) when using GFAP in the Mild (Low Risk) group. The specificity was 0.20 (0.15–0.28), PPV was 0.18 (0.13–0.25), and NPV was 0.94 (0.80–0.99; see Supplementary Table S1).

## Discussion

There is a growing body of research supporting the use of blood biomarkers for detecting intracranial CT abnormalities in patients with acute TBIs, but their integration into clinical practice has only just begun.^7,29^ The ALERT-TBI study that led to the first FDA-approved biomarker test for the acute management of patients with mTBIs, although considered pivotal, has received criticism for its lack of comparison with clinical decision rules, especially the Scandinavian Guidelines with S100B.^15,30^ We addressed this gap in the literature by combining GFAP with the clinical variables included in the Scandinavian Guidelines and examining the sensitivity and specificity of the Scandinavian Guidelines for detecting “low-risk” TBI patients who have traumatic CT abnormalities.

The modified Scandinavian guidelines with GFAP had a sensitivity of 0.94 (0.77–0.99) and a specificity of 0.20 (0.15–0.28) in detecting traumatic CT abnormalities within the imaged patients in the whole sample (*n* = 197). PPV and NPV were 0.18 (0.13–0.25) and 0.94 (0.80-0.99), respectively. In comparison, the performance of the Scandinavian Guidelines with S100B was almost identical in our previous publication (sensitivity 0.94, specificity 0.19, and PPV and NPV 0.18 and 0.94, respectively)—albeit with a larger sample.^22^ These findings suggest that the Scandinavian Guidelines perform in comparable ways using S100B and GFAP—at least in the present sample. Within the Mild (Low Risk) group (*n* = 49), a GFAP level ≥140 pg/mL was 1.0 (0.40–1.00) sensitive and 0.34 (0.19–0.53) specific for traumatic CT abnormalities, with an NPV and PPV of 1.0 (0.68–1.00) and 0.16 (0.05–0.37), respectively. Incorporating GFAP into a guideline-based subgroup of patients may have led to lowering the number of false-positive test results, compared to relying solely on the biomarker concentrations in a broad spectrum of TBI patients. GFAP levels in blood may be elevated because of age or pre-existing comorbidities in TBI patients, even in cases without traumatic CT abnormalities or in patients with no TBIs.^31–34^ By using elevated GFAP levels as an indication for imaging only in the pre-specified subgroup of patients and excluding the patients less likely to require a head CT from biomarker measurements, the probability of a false-positive test result is reduced.

In our subanalyses, we tried extending the use of biomarker measurements to the lowest risk group, which is the Minimal TBI group as defined by the Scandinavian Guidelines. According to the guidelines, patients in this group could be discharged home without imaging or blood sampling. However, our previous results revealed that 2 patients in this group had traumatic CT abnormalities on their head scans. If P-GFAP had been used in this group, it would have indicated the need for imaging in these cases. Nevertheless, this expansion of biomarker usage would have led to an additional 24 patients (8% of the total sample of 296) undergoing imaging if the elevated GFAP levels had been used in a clinical pathway within the Minimal TBI group. The ALERT-TBI study reported greater sensitivity and specificity for the FDA-approved biomarker test compared to our results, but the study sample only consisted of patients with TBIs who had undergone a head CT.^14^ This may have resulted in an enhancement of specificity, because the patients pre-determined not to need a head CT had been excluded. In contrast, our study design was prospective and S100B values may have influenced the clinicians' decision whether to refer a patient for a head CT, possibly resulting in the inclusion of participants with higher biomarker results who would have been excluded in a study design similar to ALERT-TBI.

S100B is the first biomarker to be included in a clinical decision rule for the acute management of TBI patients in the ED.^9^ The guideline is used in Europe, but the use of S100B is not recommended by either the American College of Emergency Physicians^3^ or the Eastern Association for the Surgery of Trauma^35^ for pre-head CT screening in patients with TBIs. GFAP has been shown to outperform S100B in several studies when studied as a sole predictor for traumatic CT abnormalities in patients with mTBIs,^15,18,19,36^ but a CENTER-TBI study was the first to assess the added value of combining the biomarkers with a wide range of clinical variables.^15^ That study used a previously developed clinical decision rule variable combination that was not based on the Scandinavian Guidelines; therefore, it differed somewhat from the variables used in our study by including additional risk factors, such as high-risk injury mechanisms, retrograde and post-traumatic amnesia, headache, intoxication, significant injury above clavicles, and deterioration of the GCS score. Unlike the Scandinavian Guidelines, antiplatelet medication in older adults or shunt-treated hydrocephalus were not considered as risk factors. The results of that study showed comparable discriminative ability of GFAP compared to S100B (area under the curve of 0.92 vs. 0.90, respectively). Their study did not implement any cut-off levels in the analyses; and thus, the biomarkers' performances could not be assessed further.

We extended the time limit for blood sampling from 6 h post-injury to 24 h post-injury because of the slower kinetics of GFAP compared to S100B in mTBI.^21,36^ Theoretically, the longer half-life of GFAP could make its use more efficient than S100B in the acute management of mTBI, allowing a longer measurement time window in EDs. On the other hand, the slightly slower kinetics of GFAP may influence its accuracy in very early measurement. There may be value in each biomarker at different times post-injury.

Our study has limitations, the most notable being the small sample size and the limited number of abnormal head CTs among patients with P-GFAP results in the Mild (Low Risk) group. This limitation is evident statistically, for example, in the CI for sensitivity for P-GFAP in the Mild (Low Risk) group. Future research is needed to replicate these results in a larger sample. Additionally, we did not test any other cutoffs for GFAP in our analyses in addition to the pre-selected cutoff. The cut-off selection was based on a study using a biomarker assay on the same instrument in the same laboratory that reported exploratory cutoffs for GFAP.^26^ The cutoff used was much higher than that used in another study combining serum GFAP with clinical decision rules (i.e., 30 pg/mL).^37^ However, the differences in biomarker assays between the study and ours make the lower cutoff inapplicable in our sample. Serum and plasma GFAP levels are highly correlated, but there are assay-dependent differences between the overall plasma and serum levels of GFAP in the same patients.^25,38^ This has resulted in plasma GFAP levels being much higher than serum GFAP levels in our study sample (see Supplementary Tables S7 and S8).

## Conclusion

GFAP is often considered to be the best emerging blood biomarker for head CT abnormalities in patients with head and brain injuries,^16^ and our findings suggest that when included in the subgroup of patients defined by the Scandinavian Guidelines, the biomarker can identify patients with CT abnormalities with a low but reasonable level of specificity. Future researchers should seek to replicate these findings and continue exploring the potential utility of the biomarkers.

## Supplementary Material

Supplemental data

Supplemental data

Supplemental data

Supplemental data

Supplemental data

Supplemental data

Supplemental data

Supplemental data

Supplemental data

Supplemental data

## Transparency, Rigor, and Reproducibility Summary

This study was not pre-registered. It involved secondary analyses of a study designed to validate the Scandinavian Guidelines for Initial Management of Minimal, Mild, and Moderate Head Injuries in Adults.^22^ The analysis plan was not pre-registered, but it was pre-specified to follow the analysis plan of the previous study.^22^ A total of 3067 patients were treated in the ED during the study period, and 325 gave written consent to participate. Patients presenting to the ED over 24 h from injury were excluded, leaving 296 patients. Head CT scans were completed on 197 subjects. Blood samples were collected from 287 patients, and storage samples for further analyses were stored from 204 patients. In the Mild (Low Risk) group, plasma GFAP results were available for 49 patients. Though the study protocol instructed the collection of initial blood samples and storage samples from all participants, because of the pragmatic study design blood samples were not always collected according to the intended protocol. The primary outcome (i.e., presence of acute traumatic finding on head CT) was assessed in the 36 of 49 (73.5%) patients who had undergone CT imaging. Venous blood samples were acquired between November 2015 and November 2016 in the Tampere University Hospital ED. The samples processed using centrifugation at room temperature and a part of the serum were analyzed for a previous study.^22^ The remaining samples were stored in Eppendorf tubes and immediately frozen at −70°C for future use. Samples were transferred to Mölndal, Sweden in 20 kg of dry ice by a courier for further analyses. Plasma GFAP levels were determined using the GFAP Discovery Kit (Quanterix, Billerica, MA) on an HD-1 Simoa instrument according to instructions from the manufacturer (Quanterix). Fluid biomarker measurements were performed by investigators blinded to clinical characteristics of the participants. The lower limit of detection was 0.211 pg/mL, and the lower limit of quantification was 0.686 pg/mL. Deidentified data from this study are available in the Supplementary Material and Supplementary Tables S3, S4, and S9.

## Supplementary Material

Supplementary Table S8

[Supplementary-material SD10]

## Abbreviations Used

CI: confidence interval
CT: computed tomography
ED: emergency department
FDA: U.S. Food and Drug Administration
GCS: Glasgow Coma Scale
GFAP: plasma glial fibrillary acidic protein
mTBI: mild traumatic brain injury
NPV: negative predictive value
P-GFAP: plasma GFAP
PPV: positive predictive value
S100B: S100 astroglial calcium-binding protein B
SD: standard deviation
TBI: traumatic brain injury
