# Supplementary material for: Preliminary Evaluation of the Scandinavian Guidelines for Initial Management of Minimal, Mild, and Moderate Head Injuries with Glial Fibrillary Acidic Protein
Source: Neurotrauma Rep. 2024 Jan 16;5(1):50–60. doi: 10.1089/neur.2023.0077 (PMC10797168; doi:10.1089/neur.2023.0077)
Supplement: Supplemental data [file Suppl_FigureS1.docx]

# Supplementary Figure 1. Patients divided into subgroups according to the Scandinavian guidelines with GFAP.

*
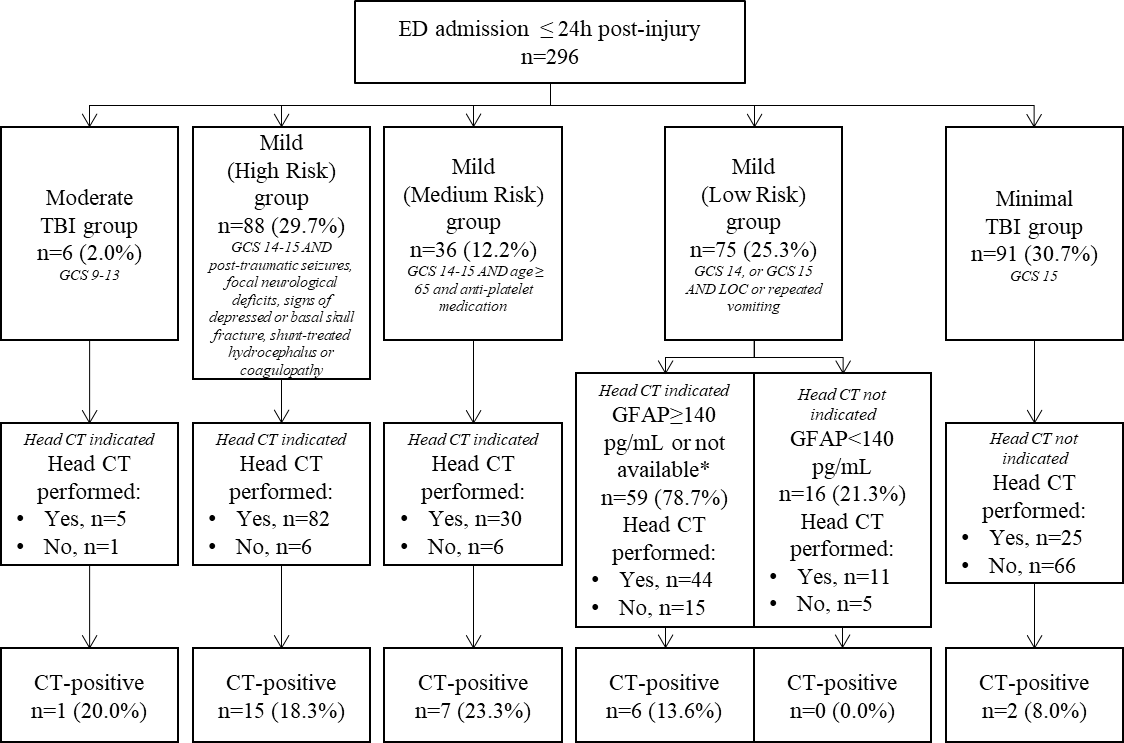
*

*Note.* ED, Emergency department; TBI, traumatic brain injury; GCS, Glasgow Coma Scale; GFAP, glial fibrillary acidic protein; CT, computed tomography.

* In the Mild (Low Risk) group, 26 (34.7%) patients did not have available plasma GFAP results because storage samples were not always collected despite being required by the original S100B validation study protocol. These patients were indicated for a head CT according to the guidelines, because the Scandinavian guidelines recommends imaging all the patients in the Mild (Low Risk) group that do not have available biomarker results. Of those patients, 19/26 (73.1%) were imaged and two had traumatic abnormalities on their head CT.
