# Supplementary material for: Preliminary Evaluation of the Scandinavian Guidelines for Initial Management of Minimal, Mild, and Moderate Head Injuries with Glial Fibrillary Acidic Protein
Source: Neurotrauma Rep. 2024 Jan 16;5(1):50–60. doi: 10.1089/neur.2023.0077 (PMC10797168; doi:10.1089/neur.2023.0077)
Supplement: Supplemental data [file Suppl_TableS2.docx]

# Supplementary Table 2. Crosstabulation of computed tomography results by the plasma GFAP levels in the Mild (Low Risk) group

|  | Computed Tomography Result | |  |
| --- | --- | --- | --- |
| Plasma GFAP | Normal | Abnormal | Total |
| <140pg/mL | 11 | 0 | 11 |
| ≥140pg/mL | 21 | 4 | 25 |
| Total | 32 | 4 | 36 |

*Note.* The sensitivity of GFAP with 140pg/mL cutoff in the Mild (Low Risk) group for detecting traumatic computed tomography (CT) abnormalities were calculated by dividing the number of patients with GFAP ≥140pg/mL and an abnormal CT result (n=4) by the total number of abnormal CT results (n=4), and the specificity by dividing the number of patients with GFAP <140pg/mL and a normal CT result (n=11) by the total number of normal CT results (n=32). The PPV were calculated by dividing the number of patients with GFAP ≥140pg/mL and an abnormal CT result (n=4) by the total number of patients with GFAP ≥140pg/mL (n=25), and the NPV by dividing the number of patients with GFAP <140pg/mL and a normal CT result (n=11) by the total number of patients with GFAP <140pg/mL (n=11). The CIs were calculated by Clinical Calculator 1 of VassarStats website (<http://vassarstats.net/clin1.html>) using the continuity corrected Newcombe-Wilson score method.

The sensitivity was 1.0 (95%CI 0.40-1.00), specificity 0.34 (95%CI 0.19-0.53), the NPV 1.0 (95%CI 0.68-1.00), and the PPV 0.16 (95%CI 0.05-0.37).
