# Supplementary material for: Preliminary Evaluation of the Scandinavian Guidelines for Initial Management of Minimal, Mild, and Moderate Head Injuries with Glial Fibrillary Acidic Protein
Source: Neurotrauma Rep. 2024 Jan 16;5(1):50–60. doi: 10.1089/neur.2023.0077 (PMC10797168; doi:10.1089/neur.2023.0077)
Supplement: Supplemental data [file Suppl_TableS5.docx]

# Supplementary Table 5. Raw data for the patients in minimal TBI group with available plasma GFAP results (n=64).

The results are sorted by imaging result and by the level of plasma GFAP.

| ID | Age | Time between injury to blood sampling  (hours) | Computed Tomography Result | Plasma  GFAP  (pg/mL) | S-S100B (ug/L) |
| --- | --- | --- | --- | --- | --- |
| T0266 | 26 | 1.9 | Normal | 42.967 | 0.150 |
| T0341 | 30 | 8.6 | Normal | 60.609 | 0.020 |
| T0183 | 51 | 4.6 | Normal | 67.179 | 0.060 |
| T0352 | 28 | 1.5 | Normal | 74.487 | 0.070 |
| T0167 | 53 | 14.3 | Normal | 79.133 | 0.030 |
| T0164 | 18 | 3.4 | Normal | 83.344 | 0.120 |
| T0249 | 54 | 2.3 | Normal | 84.755 | 0.080 |
| T0309 | 47 | 2.0 | Normal | 111.712 | 0.200 |
| T0217 | 46 | 12.9 | Normal | 145.135 | 0.070 |
| T0305 | 26 | 3.1 | Normal | 148.133 | 0.040 |
| T0203 | 60 | 3.7 | Normal | 156.475 | 0.140 |
| T0109 | 66 | 1.9 | Normal | 226.895 | 0.160 |
| T0311 | 28 | 12.3 | Normal | 312.038 | 0.050 |
| T0095 | 83 | 1.8 | Normal | 336.488 | 0.410 |
| T0176 | 22 | 3.1 | Normal | 547.874 | 0.190 |
| T0174 | 92 | 15.9 | Normal | 784.946 | 0.370 |
| T0364 | 19 | 4.9 | Normal | 1990.671 | 0.200 |
| T0230 | 88 | 4.5 | Abnormal | 886.358 | 0.050 |
| T0302 | 68 | 3.3 | Abnormal | 2857.223 | 0.440 |
| T0312 | 32 | 1.2 | Not imaged | 39.946 | 0.060 |
| T0136 | 19 | 2.3 | Not imaged | 41.089 | 0.090 |
| T0274 | 41 | 1.8 | Not imaged | 45.453 | 0.110 |
| T0111 | 26 | 1.8 | Not imaged | 48.220 | 0.240 |
| T0307 | 35 | 5.8 | Not imaged | 49.706 | 0.060 |
| T0201 | 55 | 2.0 | Not imaged | 52.247 | 0.050 |
| T0115 | 27 | 6.7 | Not imaged | 55.186 | 0.100 |
| T0146 | 41 | 6.1 | Not imaged | 61.615 | 0.070 |
| T0160 | 42 | 1.3 | Not imaged | 62.280 | 0.140 |
| T0202 | 45 | 2.6 | Not imaged | 62.432 | 0.130 |
| T0093 | 26 | 1.4 | Not imaged | 62.534 | 0.250 |
| T0097 | 55 | 12.1 | Not imaged | 71.850 | 0.060 |
| T0359 | 34 | 2.9 | Not imaged | 75.084 | 0.080 |
| T0261 | 18 | 3.7 | Not imaged | 76.597 | 0.050 |
| T0293 | 18 | 3.0 | Not imaged | 84.998 | 0.110 |
| T0252 | 32 | 0.8 | Not imaged | 90.787 | 0.910 |
| T0264 | 60 | 1.9 | Not imaged | 91.952 | 0.070 |
| T0209 | 49 | 0.8 | Not imaged | 93.216 | 0.150 |
| T0127 | 21 | 3.3 | Not imaged | 100.906 | 0.250 |
| T0130 | 27 | 10.8 | Not imaged | 102.030 | 0.080 |
| T0270 | 69 | 2.0 | Not imaged | 116.226 | 0.180 |
| T0197 | 69 | 1.2 | Not imaged | 117.080 | 0.100 |
| T0296 | 61 | 4.8 | Not imaged | 117.983 | 0.060 |
| T0162 | 56 | 16.5 | Not imaged | 128.377 | 0.260 |
| T0358 | 35 | 3.3 | Not imaged | 140.026 | 0.070 |
| T0365 | 18 | 2.5 | Not imaged | 177.203 | 0.040 |
| T0145 | 51 | 7.2 | Not imaged | 188.012 | 0.100 |
| T0084 | 81 | 3.1 | Not imaged | 191.730 | 0.100 |
| T0114 | 91 | 2.4 | Not imaged | 192.092 | 0.140 |
| T0119 | 72 | 3.3 | Not imaged | 207.262 | 0.110 |
| T0288 | 84 | 2.6 | Not imaged | 230.510 | 0.110 |
| T0234 | 23 | 1.4 | Not imaged | 249.934 | 0.390 |
| T0327 | 68 | 1.8 | Not imaged | 281.190 | 0.170 |
| T0259 | 25 | 4.6 | Not imaged | 288.392 | 0.080 |
| T0133 | 87 | 4.3 | Not imaged | 288.561 | 0.100 |
| T0329 | 49 | 4.0 | Not imaged | 326.559 | 0.050 |
| T0110 | 70 | 2.3 | Not imaged | 326.791 | 0.110 |
| T0185 | 47 | 4.3 | Not imaged | 339.087 | 0.110 |
| T0208 | 50 | 2.8 | Not imaged | 346.453 | 0.100 |
| T0129 | 36 | 2.5 | Not imaged | 348.436 | 0.130 |
| T0086 | 76 | 1.8 | Not imaged | 375.118 | 0.210 |
| T0233 | 39 | 3.8 | Not imaged | 415.445 | 0.070 |
| T0088 | 72 | 3.8 | Not imaged | 631.256 | 0.150 |
| T0141 | 87 | 2.1 | Not imaged | 748.872 | 0.400 |
| T0148 | 75 | 7.2 | Not imaged | 4419.954 | 0.210 |
