# Supplementary material for: Preliminary Evaluation of the Scandinavian Guidelines for Initial Management of Minimal, Mild, and Moderate Head Injuries with Glial Fibrillary Acidic Protein
Source: Neurotrauma Rep. 2024 Jan 16;5(1):50–60. doi: 10.1089/neur.2023.0077 (PMC10797168; doi:10.1089/neur.2023.0077)
Supplement: Supplemental data [file Suppl_TableS6.docx]

# Supplementary Table 6. Crosstabulation of computed tomography results by the plasma GFAP levels in the combined Minimal and Mild (Low Risk) group

|  | Computed Tomography Result | |  |
| --- | --- | --- | --- |
| Plasma GFAP | Normal | Abnormal | Total |
| <140pg/mL | 19 | 0 | 19 |
| ≥140pg/mL | 30 | 6 | 36 |
| Total | 49 | 6 | 55 |

*Note.* The sensitivity of GFAP with 140pg/mL cutoff in the combined group for detecting traumatic computed tomography (CT) abnormalities were calculated by dividing the number of patients with GFAP ≥140pg/mL and an abnormal CT result (n=6) by the total number of abnormal CT results (n=6), and the specificity by dividing the number of patients with GFAP <140pg/mL and a normal CT result (n=19) by the total number of normal CT results (n=49). The PPV were calculated by dividing the number of patients with GFAP ≥140pg/mL and an abnormal CT result (n=6) by the total number of patients with GFAP ≥140pg/mL (n=36), and the NPV by dividing the number of patients with GFAP <140pg/mL and a normal CT result (n=19) by the total number of patients with GFAP <140pg/mL (n=19). The CIs were calculated by Clinical Calculator 1 of VassarStats website (<http://vassarstats.net/clin1.html>) using the continuity corrected Newcombe-Wilson score method.

The sensitivity was 1.0 (95%CI 0.52-1.00), specificity 0.39 (95%CI 0.26-0.54), the NPV 1.0 (95%CI 0.79-1.00), and the PPV 0.17 (95%CI 0.07-0.33).
