# Supplementary material for: Preliminary Evaluation of the Scandinavian Guidelines for Initial Management of Minimal, Mild, and Moderate Head Injuries with Glial Fibrillary Acidic Protein
Source: Neurotrauma Rep. 2024 Jan 16;5(1):50–60. doi: 10.1089/neur.2023.0077 (PMC10797168; doi:10.1089/neur.2023.0077)
Supplement: Supplemental data [file Suppl_TableS1.docx]

# Supplementary Table 1. Crosstabulation of computed tomography results by the modified guideline indication for imaging

|  | Computed Tomography Result | |  |
| --- | --- | --- | --- |
| Guideline indication | Normal | Abnormal | Total |
| Imaging not indicated | 34 | 2 | 36 |
| Imaging indicated | 132 | 29 | 161 |
| Total | 166 | 31 | 197 |

*Note.* The modified guideline sensitivity for detecting traumatic computed tomography (CT) abnormalities were calculated by dividing the number of patients with an indication for imaging and an abnormal CT result (n=29) by the total number of abnormal CT results (n=31), and the specificity by dividing the number of patients without an indication for imaging and a normal CT result (n=34) by the total number of normal CT results (n=166). The positive predictive value (PPV) were calculated by dividing the number of patients with an indication for imaging and an abnormal CT result (n=29) by the total number of patients with an indication for imaging (n=161), and the negative predictive value (NPV) by dividing the number of patients without an indication for imaging and a normal CT result (n=34) by the total number of patients without an indication for imaging (n=36). The confidence intervals (CI) were calculated by Clinical Calculator 1 of VassarStats website (<http://vassarstats.net/clin1.html>) using the continuity corrected Newcombe-Wilson score method.

The sensitivity was 0.94 (95%CI 0.77-0.99), specificity 0.20 (95%CI 0.15-0.28), the PPV 0.18 (95%CI 0.13-0.25), and the NPV 0.94 (95%CI 0.80-0.99).
