# Supplementary material for: Preliminary Evaluation of the Scandinavian Guidelines for Initial Management of Minimal, Mild, and Moderate Head Injuries with Glial Fibrillary Acidic Protein
Source: Neurotrauma Rep. 2024 Jan 16;5(1):50–60. doi: 10.1089/neur.2023.0077 (PMC10797168; doi:10.1089/neur.2023.0077)
Supplement: Supplemental data [file Suppl_TableS7.docx]

# Supplementary Table 7. Six biomarkers with cases sorted by plasma GFAP (n=49)

The subjects were sorted by CT result and by level of **plasma GFAP**.

| ID | Age | Time between injury to blood sampling (hours) | Time between injury to head CT (hours) | Computed Tomography Result | P-GFAP (pg/mL) | S-GFAP (pg/mL) | S-NFL (pg/mL) | S-Tau (pg/mL) | S-UCH-L1 (pg/mL) | S-S100B (ug/L) |
| --- | --- | --- | --- | --- | --- | --- | --- | --- | --- | --- |
| T0291 | 20.0 | 15.1 | 15.1 | Normal | 37.018 | 37.718 | 2.618 | 0.547 | N/A | 0.270 |
| T0276 | 25.0 | 1.7 | 1.8 | Normal | 37.986 | 47.665 | 5.645 | 2.096 | 13.305 | 0.180 |
| T0284 | 26.0 | 1.7 | 3.9 | Normal | 50.598 | 33.643 | 7.153 | 0.236 | 163.726 | 0.090 |
| T0344 | 43.0 | 1.8 | 2.5 | Normal | 60.465 | 30.204 | 6.561 | 0.553 | 14.130 | 0.230 |
| T0346 | 32.0 | 3.2 | 3.5 | Normal | 63.258 | 44.801 | 5.217 | 1.010 | 9.790 | 0.070 |
| T0300 | 52.0 | 3.9 | 4.4 | Normal | 80.445 | 78.844 | 6.869 | 0.941 | 84.976 | 0.090 |
| T0349 | 24.0 | 6.7 | 6.7 | Normal | 90.768 | 50.291 | 8.080 | 1.234 | 18.413 | 0.070 |
| T0144 | 70.0 | 5.1 | 6.2 | Normal | 95.687 | 98.566 | 10.392 | 0.814 | 127.998 | 0.090 |
| T0304 | 21.0 | 2.2 | 2.4 | Normal | 100.349 | 79.972 | 9.016 | 2.330 | 107.013 | 0.240 |
| T0143 | 50.0 | 4.3 | 5.8 | Normal | 108.217 | 111.052 | 24.774 | 3.142 | 13.099 | 0.160 |
| T0247 | 49.0 | 4.6 | 5.7 | Normal | 137.176 | 73.528 | 11.425 | 0.891 | 58.485 | 0.120 |
| T0255 | 50.0 | 3.5 | 4.9 | Normal | 153.905 | 148.051 | 7.511 | 2.893 | 85.585 | 0.340 |
| T0170 | 47.0 | 3.9 | 4.2 | Normal | 154.109 | 74.944 | 8.790 | 1.193 | 17.710 | 0.090 |
| T0262 | 68.0 | 0.8 | 4.1 | Normal | 155.255 | 170.991 | 31.041 | 0.533 | 20.162 | 0.120 |
| T0171 | 48.0 | 6.0 | 6.0 | Normal | 167.894 | 123.060 | 10.202 | 1.096 | 37.724 | 0.120 |
| T0168 | 48.0 | 1.5 | 1.7 | Normal | 201.716 | 102.805 | 6.869 | 0.707 | 24.829 | 0.070 |
| T0303 | 21.0 | 1.8 | 4.6 | Normal | 211.310 | 148.711 | 4.830 | 1.020 | 59.712 | 0.100 |
| T0214 | 65.0 | 2.4 | 4.7 | Normal | 212.542 | 135.817 | 14.976 | 1.004 | 24.390 | 0.150 |
| T0179 | 75.0 | 1.4 | 1.8 | Normal | 272.348 | 166.290 | 17.919 | 2.019 | 65.977 | 0.120 |
| T0350 | 22.0 | 3.3 | 2.5 | Normal | 293.384 | 110.861 | 7.127 | 2.350 | 70.661 | 0.280 |
| T0281 | 75.0 | 3.6 | 5.0 | Normal | 386.356 | 504.937 | 23.078 | 3.306 | 26.802 | 0.230 |
| T0182 | 24.0 | 2.0 | 2.2 | Normal | 439.319 | 298.989 | 6.213 | 1.936 | 54.699 | 0.180 |
| T0219 | 58.0 | 4.3 | 3.0 | Normal | 513.266 | 375.913 | 19.074 | 0.427 | 17.258 | 0.120 |
| T0177 | 94.0 | 2.7 | 4.6 | Normal | 517.067 | 272.902 | 31.277 | 0.894 | 46.483 | 0.700 |
| T0163 | 77.0 | 1.7 | 3.1 | Normal | 593.990 | 477.162 | 18.281 | 1.287 | 23.982 | 0.450 |
| T0353 | 61.0 | 3.5 | 6.8 | Normal | 795.052 | 434.286 | 9.055 | 1.531 | 107.582 | 0.270 |
| T0134 | 18.0 | 2.3 | 0.8 | Normal | 901.565 | 908.514 | 14.989 | 2.333 | 66.975 | 0.200 |
| T0272 | 29.0 | 4.2 | 4.8 | Normal | 1025.266 | 1069.163 | 5.573 | 0.991 | 21.171 | 0.080 |
| T0268 | 69.0 | 3.1 | 3.1 | Normal | 1283.277 | 218.441 | 15.132 | 0.846 | 10.236 | 0.080 |
| T0306 | 41.0 | 3.9 | 5.9 | Normal | 1353.578 | 1337.487 | 11.693 | 1.105 | 31.969 | 0.140 |
| T0211 | 85.0 | 16.1 | 17.5 | Normal | 1614.338 | 1011.651 | 112.264 | 0.891 | 58.160 | 0.070 |
| T0190 | 47.0 | 2.8 | 3.4 | Normal | 8271.513 | 345.550 | 9.068 | 3.215 | 11.325 | 0.190 |
| T0351 | 72.0 | 4.3 | 8.3 | Abnormal | 437.059 | 349.994 | 19.463 | 0.708 | 15.073 | 0.300 |
| T0189 | 61.0 | 5.1 | 4.8 | Abnormal | 1490.067 | 846.644 | 73.089 | 1.968 | 48.201 | 0.110 |
| T0200 | 42.0 | 2.1 | 3.9 | Abnormal | 3768.306 | 2885.245 | 8.486 | 1.373 | 71.712 | 0.330 |
| T0215 | 72.0 | 3.8 | 4.2 | Abnormal | 4296.402 | 2212.887 | 19.594 | 1.021 | 100.462 | 0.160 |
| T0083 | 22.0 | 10.7 | N/A | Not imaged | 93.977 | 71.144 | 5.612 | 0.971 | 30.330 | 0.080 |
| T0122 | 30.0 | 7.6 | N/A | Not imaged | 97.173 | 89.862 | 4.060 | 2.013 | 58.306 | 0.270 |
| T0226 | 19.0 | 5.6 | N/A | Not imaged | 101.733 | 55.359 | 4.252 | 0.723 | 5.366 | 0.060 |
| T0298 | 67.0 | 1.4 | N/A | Not imaged | 109.056 | 81.064 | 18.733 | 0.201 | 16.985 | 0.240 |
| T0256 | 71.0 | 5.3 | N/A | Not imaged | 138.596 | 144.934 | 64.729 | 1.954 | 74.616 | 0.100 |
| T0147 | 24.0 | 2.1 | N/A | Not imaged | 142.372 | 115.498 | 6.907 | 1.820 | 109.893 | 0.070 |
| T0154 | 72.0 | 2.6 | N/A | Not imaged | 158.409 | 116.269 | 17.863 | 0.612 | 9.669 | 0.050 |
| T0091 | 55.0 | 2.2 | N/A | Not imaged | 173.236 | 116.983 | 13.456 | 0.828 | N/A | 0.260 |
| T0336 | 48.0 | 1.2 | N/A | Not imaged | 181.202 | 135.939 | 14.990 | 0.302 | 35.222 | 0.200 |
| T0223 | 58.0 | 2.1 | N/A | Not imaged | 205.098 | 125.523 | 13.586 | 1.377 | 26.037 | 0.090 |
| T0275 | 61.0 | 0.5 | N/A | Not imaged | 278.824 | 272.143 | 15.824 | 0.705 | 59.722 | 0.110 |
| T0248 | 19.0 | 4.6 | N/A | Not imaged | 283.592 | 147.792 | 4.611 | 0.380 | 43.149 | 0.040 |
| T0221 | 25.0 | 10.8 | N/A | Not imaged | 1351.705 | 621.943 | 4.087 | 1.048 | 20.229 | 0.080 |

Note: Future researchers might be interested in other biomarker values in these patients. The following description was reprinted from Iverson GL. Minkkinen M. Karr JE. Berghem K. Zetterberg H. Blennow K. Posti JP. Luoto TM. Examining four blood biomarkers for the detection of acute intracranial abnormalities following mild traumatic brain injury in older adults. Front Neurol. 2022 Nov 22;13:960741. doi: 10.3389/fneur.2022.960741. PMID: 36484020; PMCID: PMC9723459. ‘Venous blood samples were collected within 12 hours of injury. Blood samples were centrifuged for 10 minutes at 10.000 rpm at room temperature. Part of the serum was analyzed at Tampere University Hospital (Tampere. Finland) as part of the hospital laboratory’s on-call services for a prior study.(61) The remaining serum was stored in Eppendorf tubes and immediately frozen at -70°C for future use. The blood samples were collected in Tampere between November 2015 and November 2016. Approximately two years later the serum was sent to the Sahlgrenska University Hospital (research laboratory) in Mölndal Sweden for analysis. All the serum samples were transferred in 20 kilograms of dry ice from Tampere to Mölndal. The samples analyzed in Mölndal underwent one cycle of freezing and thawing. The serum samples were analyzed in March of 2018 using the Quanterix Simoa 4-plex assay on a Simoa HD-1 analyzer according to the manufacturer’s instructions (Quanterix. Billerica. MA). The 4-plex assay measures four protein biomarkers in blood: GFAP. NF-L. t-tau. and UCH-L1. For GFAP. the lower limit of quantification (LLOQ) was 0.467 pg/mL. the lower limit of detection (LLOD) was 0.221 pg/mL. and the calibration range was 0–1000 pg/mL. For NF-L. LLOQ was 0.241 pg/mL. LLOD was 0.104 pg/mL. and the calibration range was 0–500 pg/mL. For t-tau. LLOQ was 0.053 pg/mL. LLOD was 0.024 pg/mL. and the calibration range was 0–100 pg/mL. For UCH-L1. LLOQ was 5.45 pg/mL. LLOD 1.74 pg/mL. and the calibration range was 0-10 ng/mL. The mean interval in which the serum was frozen was 23.9 months (SD=2.9. Range=17-27). The laboratory technicians performing the analyses were blinded to the clinical data.’

The following description is from Huebschmann NA. Luoto TM. Karr JE. Berghem K. Blennow K. Zetterberg H. Ashton NJ. Simrén J. Posti JP. Gill JM. Iverson GL. Comparing Glial Fibrillary Acidic Protein (GFAP) in Serum and Plasma Following Mild Traumatic Brain Injury in Older Adults. Front Neurol. 2020 Sep 18;11:1054. doi: 10.3389/fneur.2020.01054. PMID: 33071938; PMCID: PMC7530818. ‘Plasma GFAP levels were analyzed on September 14-15. 2019. again in Mölndal. Sweden using the GFAP Discovery Kit (Quanterix. Billerica. MA) on an HD-1 Simoa instrument according to instructions from the manufacturer (Quanterix. Billerica. MA). The lower limit of detection for GFAP was 0.211 pg/mL and the lower limit of quantification was 0.686 pg/mL. Calibrators were run in duplicates while samples were run in singlicates. Samples were run with a 4-fold dilution and results have been compensated for this dilution. Two internal quality control samples were run in duplicates in the beginning and end of each run. For a quality control sample with a concentration of 76.3 pg/mL. repeatability was 7.6% and intermediate precision was 11.3%. whereas for a quality control sample with a concentration of 204.2 pg/mL. repeatability was 6.8% and intermediate precision was 12.8%.’
