# Supplementary material for: Preliminary Evaluation of the Scandinavian Guidelines for Initial Management of Minimal, Mild, and Moderate Head Injuries with Glial Fibrillary Acidic Protein
Source: Neurotrauma Rep. 2024 Jan 16;5(1):50–60. doi: 10.1089/neur.2023.0077 (PMC10797168; doi:10.1089/neur.2023.0077)
Supplement: Supplemental data [file Suppl_TableS8.docx]

# Supplementary Table 8. Six biomarkers with cases sorted by serum GFAP (n=49)

The subjects were sorted by CT result and by level of **serum GFAP**.

| ID | Age | Time between injury to blood sampling (hours) | Time between injury to head CT (hours) | Computed Tomography Result | P-GFAP (pg/mL) | S-GFAP (pg/mL) | S-NFL  (pg/mL) | S-Tau  (pg/mL) | S-UCH-L1  (pg/mL) | S100B  (ug/L) |
| --- | --- | --- | --- | --- | --- | --- | --- | --- | --- | --- |
| T0344 | 43.0 | 1.8 | 2.5 | Normal | 60.465 | 30.204 | 6.561 | 0.553 | 14.130 | 0.230 |
| T0284 | 26.0 | 1.7 | 3.9 | Normal | 50.598 | 33.643 | 7.153 | 0.236 | 163.726 | 0.090 |
| T0291 | 20.0 | 15.1 | 15.1 | Normal | 37.018 | 37.718 | 2.618 | 0.547 | N/A | 0.270 |
| T0346 | 32.0 | 3.2 | 3.5 | Normal | 63.258 | 44.801 | 5.217 | 1.010 | 9.790 | 0.070 |
| T0276 | 25.0 | 1.7 | 1.8 | Normal | 37.986 | 47.665 | 5.645 | 2.096 | 13.305 | 0.180 |
| T0349 | 24.0 | 6.7 | 6.7 | Normal | 90.768 | 50.291 | 8.080 | 1.234 | 18.413 | 0.070 |
| T0247 | 49.0 | 4.6 | 5.7 | Normal | 137.176 | 73.528 | 11.425 | 0.891 | 58.485 | 0.120 |
| T0170 | 47.0 | 3.9 | 4.2 | Normal | 154.109 | 74.944 | 8.790 | 1.193 | 17.710 | 0.090 |
| T0300 | 52.0 | 3.9 | 4.4 | Normal | 80.445 | 78.844 | 6.869 | 0.941 | 84.976 | 0.090 |
| T0304 | 21.0 | 2.2 | 2.4 | Normal | 100.349 | 79.972 | 9.016 | 2.330 | 107.013 | 0.240 |
| T0144 | 70.0 | 5.1 | 6.2 | Normal | 95.687 | 98.566 | 10.392 | 0.814 | 127.998 | 0.090 |
| T0168 | 48.0 | 1.5 | 1.7 | Normal | 201.716 | 102.805 | 6.869 | 0.707 | 24.829 | 0.070 |
| T0350 | 22.0 | 3.3 | 2.5 | Normal | 293.384 | 110.861 | 7.127 | 2.350 | 70.661 | 0.280 |
| T0143 | 50.0 | 4.3 | 5.8 | Normal | 108.217 | 111.052 | 24.774 | 3.142 | 13.099 | 0.160 |
| T0171 | 48.0 | 6.0 | 6.0 | Normal | 167.894 | 123.060 | 10.202 | 1.096 | 37.724 | 0.120 |
| T0214 | 65.0 | 2.4 | 4.7 | Normal | 212.542 | 135.817 | 14.976 | 1.004 | 24.390 | 0.150 |
| T0255 | 50.0 | 3.5 | 4.9 | Normal | 153.905 | 148.051 | 7.511 | 2.893 | 85.585 | 0.340 |
| T0303 | 21.0 | 1.8 | 4.6 | Normal | 211.310 | 148.711 | 4.830 | 1.020 | 59.712 | 0.100 |
| T0179 | 75.0 | 1.4 | 1.8 | Normal | 272.348 | 166.290 | 17.919 | 2.019 | 65.977 | 0.120 |
| T0262 | 68.0 | 0.8 | 4.1 | Normal | 155.255 | 170.991 | 31.041 | 0.533 | 20.162 | 0.120 |
| T0268 | 69.0 | 3.1 | 3.1 | Normal | 1283.277 | 218.441 | 15.132 | 0.846 | 10.236 | 0.080 |
| T0177 | 94.0 | 2.7 | 4.6 | Normal | 517.067 | 272.902 | 31.277 | 0.894 | 46.483 | 0.700 |
| T0182 | 24.0 | 2.0 | 2.2 | Normal | 439.319 | 298.989 | 6.213 | 1.936 | 54.699 | 0.180 |
| T0190 | 47.0 | 2.8 | 3.4 | Normal | 8271.513 | 345.550 | 9.068 | 3.215 | 11.325 | 0.190 |
| T0219 | 58.0 | 4.3 | 3.0 | Normal | 513.266 | 375.913 | 19.074 | 0.427 | 17.258 | 0.120 |
| T0353 | 61.0 | 3.5 | 6.8 | Normal | 795.052 | 434.286 | 9.055 | 1.531 | 107.582 | 0.270 |
| T0163 | 77.0 | 1.7 | 3.1 | Normal | 593.990 | 477.162 | 18.281 | 1.287 | 23.982 | 0.450 |
| T0281 | 75.0 | 3.6 | 5.0 | Normal | 386.356 | 504.937 | 23.078 | 3.306 | 26.802 | 0.230 |
| T0134 | 18.0 | 2.3 | 0.8 | Normal | 901.565 | 908.514 | 14.989 | 2.333 | 66.975 | 0.200 |
| T0211 | 85.0 | 16.1 | 17.5 | Normal | 1614.338 | 1011.651 | 112.264 | 0.891 | 58.160 | 0.070 |
| T0272 | 29.0 | 4.2 | 4.8 | Normal | 1025.266 | 1069.163 | 5.573 | 0.991 | 21.171 | 0.080 |
| T0306 | 41.0 | 3.9 | 5.9 | Normal | 1353.578 | 1337.487 | 11.693 | 1.105 | 31.969 | 0.140 |
| T0351 | 72.0 | 4.3 | 8.3 | Abnormal | 437.059 | 349.994 | 19.463 | 0.708 | 15.073 | 0.300 |
| T0189 | 61.0 | 5.1 | 4.8 | Abnormal | 1490.067 | 846.644 | 73.089 | 1.968 | 48.201 | 0.110 |
| T0215 | 72.0 | 3.8 | 4.2 | Abnormal | 4296.402 | 2212.887 | 19.594 | 1.021 | 100.462 | 0.160 |
| T0200 | 42.0 | 2.1 | 3.9 | Abnormal | 3768.306 | 2885.245 | 8.486 | 1.373 | 71.712 | 0.330 |
| T0226 | 19.0 | 5.6 | N/A | Not imaged | 101.733 | 55.359 | 4.252 | 0.723 | 5.366 | 0.060 |
| T0083 | 22.0 | 10.7 | N/A | Not imaged | 93.977 | 71.144 | 5.612 | 0.971 | 30.330 | 0.080 |
| T0298 | 67.0 | 1.4 | N/A | Not imaged | 109.056 | 81.064 | 18.733 | 0.201 | 16.985 | 0.240 |
| T0122 | 30.0 | 7.6 | N/A | Not imaged | 97.173 | 89.862 | 4.060 | 2.013 | 58.306 | 0.270 |
| T0147 | 24.0 | 2.1 | N/A | Not imaged | 142.372 | 115.498 | 6.907 | 1.820 | 109.893 | 0.070 |
| T0154 | 72.0 | 2.6 | N/A | Not imaged | 158.409 | 116.269 | 17.863 | 0.612 | 9.669 | 0.050 |
| T0091 | 55.0 | 2.2 | N/A | Not imaged | 173.236 | 116.983 | 13.456 | 0.828 | N/A | 0.260 |
| T0223 | 58.0 | 2.1 | N/A | Not imaged | 205.098 | 125.523 | 13.586 | 1.377 | 26.037 | 0.090 |
| T0336 | 48.0 | 1.2 | N/A | Not imaged | 181.202 | 135.939 | 14.990 | 0.302 | 35.222 | 0.200 |
| T0256 | 71.0 | 5.3 | N/A | Not imaged | 138.596 | 144.934 | 64.729 | 1.954 | 74.616 | 0.100 |
| T0248 | 19.0 | 4.6 | N/A | Not imaged | 283.592 | 147.792 | 4.611 | 0.380 | 43.149 | 0.040 |
| T0275 | 61.0 | 0.5 | N/A | Not imaged | 278.824 | 272.143 | 15.824 | 0.705 | 59.722 | 0.110 |
| T0221 | 25.0 | 10.8 | N/A | Not imaged | 1351.705 | 621.943 | 4.087 | 1.048 | 20.229 | 0.080 |
