# Supplementary material for: Preliminary Evaluation of the Scandinavian Guidelines for Initial Management of Minimal, Mild, and Moderate Head Injuries with Glial Fibrillary Acidic Protein
Source: Neurotrauma Rep. 2024 Jan 16;5(1):50–60. doi: 10.1089/neur.2023.0077 (PMC10797168; doi:10.1089/neur.2023.0077)
Supplement: Supplemental data [file Suppl_TableS4.docx]

# Supplementary Table 4. Raw data for each subject (n=49)

The results are sorted by imaging result and by the level of plasma GFAP.

| ID | Age | Time between injury to blood sampling  (hours) | Time between injury to head CT  (hours) | Computed Tomography Result | Plasma  GFAP  (pg/mL) |
| --- | --- | --- | --- | --- | --- |
| T0291 | 20.0 | 15.1 | 15.1 | Normal | 37.018 |
| T0276 | 25.0 | 1.7 | 1.8 | Normal | 37.986 |
| T0284 | 26.0 | 1.7 | 3.9 | Normal | 50.598 |
| T0344 | 43.0 | 1.8 | 2.5 | Normal | 60.465 |
| T0346 | 32.0 | 3.2 | 3.5 | Normal | 63.258 |
| T0300 | 52.0 | 3.9 | 4.4 | Normal | 80.445 |
| T0349 | 24.0 | 6.7 | 6.7 | Normal | 90.768 |
| T0144 | 70.0 | 5.1 | 6.2 | Normal | 95.687 |
| T0304 | 21.0 | 2.2 | 2.4 | Normal | 100.349 |
| T0143 | 50.0 | 4.3 | 5.8 | Normal | 108.217 |
| T0247 | 49.0 | 4.6 | 5.7 | Normal | 137.176 |
| T0255 | 50.0 | 3.5 | 4.9 | Normal | 153.905 |
| T0170 | 47.0 | 3.9 | 4.2 | Normal | 154.109 |
| T0262 | 68.0 | 0.8 | 4.1 | Normal | 155.255 |
| T0171 | 48.0 | 6.0 | 6.0 | Normal | 167.894 |
| T0168 | 48.0 | 1.5 | 1.7 | Normal | 201.716 |
| T0303 | 21.0 | 1.8 | 4.6 | Normal | 211.310 |
| T0214 | 65.0 | 2.4 | 4.7 | Normal | 212.542 |
| T0179 | 75.0 | 1.4 | 1.8 | Normal | 272.348 |
| T0350 | 22.0 | 3.3 | 2.5 | Normal | 293.384 |
| T0281 | 75.0 | 3.6 | 5.0 | Normal | 386.356 |
| T0182 | 24.0 | 2.0 | 2.2 | Normal | 439.319 |
| T0219 | 58.0 | 4.3 | 3.0 | Normal | 513.266 |
| T0177 | 94.0 | 2.7 | 4.6 | Normal | 517.067 |
| T0163 | 77.0 | 1.7 | 3.1 | Normal | 593.990 |
| T0353 | 61.0 | 3.5 | 6.8 | Normal | 795.052 |
| T0134 | 18.0 | 2.3 | 0.8 | Normal | 901.565 |
| T0272 | 29.0 | 4.2 | 4.8 | Normal | 1025.266 |
| T0268 | 69.0 | 3.1 | 3.1 | Normal | 1283.277 |
| T0306 | 41.0 | 3.9 | 5.9 | Normal | 1353.578 |
| T0211 | 85.0 | 16.1 | 17.5 | Normal | 1614.338 |
| T0190 | 47.0 | 2.8 | 3.4 | Normal | 8271.513 |
| T0351 | 72.0 | 4.3 | 8.3 | Abnormal | 437.059 |
| T0189 | 61.0 | 5.1 | 4.8 | Abnormal | 1490.067 |
| T0200 | 42.0 | 2.1 | 3.9 | Abnormal | 3768.306 |
| T0215 | 72.0 | 3.8 | 4.2 | Abnormal | 4296.402 |
| T0083 | 22.0 | 10.7 | - | Not imaged | 93.977 |
| T0122 | 30.0 | 7.6 | - | Not imaged | 97.173 |
| T0226 | 19.0 | 5.6 | - | Not imaged | 101.733 |
| T0298 | 67.0 | 1.4 | - | Not imaged | 109.056 |
| T0256 | 71.0 | 5.3 | - | Not imaged | 138.596 |
| T0147 | 24.0 | 2.1 | - | Not imaged | 142.372 |
| T0154 | 72.0 | 2.6 | - | Not imaged | 158.409 |
| T0091 | 55.0 | 2.2 | - | Not imaged | 173.236 |
| T0336 | 48.0 | 1.2 | - | Not imaged | 181.202 |
| T0223 | 58.0 | 2.1 | - | Not imaged | 205.098 |
| T0275 | 61.0 | 0.5 | - | Not imaged | 278.824 |
| T0248 | 19.0 | 4.6 | - | Not imaged | 283.592 |
| T0221 | 25.0 | 10.8 | - | Not imaged | 1351.705 |
