# Supplementary material for: Preliminary Evaluation of the Scandinavian Guidelines for Initial Management of Minimal, Mild, and Moderate Head Injuries with Glial Fibrillary Acidic Protein
Source: Neurotrauma Rep. 2024 Jan 16;5(1):50–60. doi: 10.1089/neur.2023.0077 (PMC10797168; doi:10.1089/neur.2023.0077)
Supplement: Supplemental data [file Suppl_TableS9.docx]

# Supplementary Table 9. Raw data for each subject (n=197)

The results are sorted by imaging result and by the level of **plasma GFAP**.

| ID | Age | Time between injury to blood sampling  (hours) | Time between injury to head CT  (hours) | Computed Tomography Result | Plasma  GFAP  (pg/mL) |
| --- | --- | --- | --- | --- | --- |
| T0291 | 20.0 | 15.1 | 11.2 | Normal | 37.018 |
| T0276 | 25.0 | 1.7 | 0.6 | Normal | 37.986 |
| T0266 | 26.0 | 1.9 | 1.4 | Normal | 42.967 |
| T0284 | 26.0 | 1.7 | 0.4 | Normal | 50.598 |
| T0344 | 43.0 | 1.8 | 1.4 | Normal | 60.465 |
| T0341 | 30.0 | 8.6 | 7.5 | Normal | 60.609 |
| T0346 | 32.0 | 3.2 | 0.7 | Normal | 63.258 |
| T0285 | 56.0 | 2.1 | 1.0 | Normal | 63.357 |
| T0183 | 51.0 | 4.6 | 2.9 | Normal | 67.179 |
| T0352 | 28.0 | 1.5 | 1.0 | Normal | 74.487 |
| T0287 | 71.0 | 2.4 | 2.1 | Normal | 77.404 |
| T0167 | 53.0 | 14.3 | 11.5 | Normal | 79.133 |
| T0300 | 52.0 | 3.9 | 1.9 | Normal | 80.445 |
| T0164 | 18.0 | 3.4 | 1.8 | Normal | 83.344 |
| T0186 | 31.0 | 20.3 | 18.3 | Normal | 84.242 |
| T0249 | 54.0 | 2.3 | 0.6 | Normal | 84.755 |
| T0349 | 24.0 | 6.7 | 3.7 | Normal | 90.768 |
| T0144 | 70.0 | 5.1 | 2.8 | Normal | 95.687 |
| T0149 | 67.0 | 1.3 | 0.3 | Normal | 99.755 |
| T0304 | 21.0 | 2.2 | 1.4 | Normal | 100.349 |
| T0299 | 58.0 | 5.3 | 0.7 | Normal | 104.124 |
| T0143 | 50.0 | 4.3 | 2.0 | Normal | 108.217 |
| T0309 | 47.0 | 2.0 | 1.1 | Normal | 111.712 |
| T0260 | 77.0 | 2.1 | 0.7 | Normal | 116.154 |
| T0339 | 86.0 | 2.9 | 0.5 | Normal | 127.461 |
| T0204 | 66.0 | 3.3 | 0.5 | Normal | 135.163 |
| T0247 | 49.0 | 4.6 | 4.4 | Normal | 137.176 |
| T0217 | 46.0 | 12.9 | 6.0 | Normal | 145.135 |
| T0305 | 26.0 | 3.1 | 0.4 | Normal | 148.133 |
| T0243 | 59.0 | 2.6 | 1.3 | Normal | 149.380 |
| T0255 | 50.0 | 3.5 | 1.8 | Normal | 153.905 |
| T0170 | 47.0 | 3.9 | 0.7 | Normal | 154.109 |
| T0262 | 68.0 | 0.8 | 0.2 | Normal | 155.255 |
| T0203 | 60.0 | 3.7 | 0.6 | Normal | 156.475 |
| T0224 | 78.0 | 6.1 | 1.3 | Normal | 156.508 |
| T0128 | 68.0 | 14.9 | 10.9 | Normal | 158.215 |
| T0171 | 48.0 | 6.0 | 0.9 | Normal | 167.894 |
| T0180 | 51.0 | 1.6 | 1.4 | Normal | 168.806 |
| T0279 | 70.0 | 3.4 | 2.6 | Normal | 178.795 |
| T0181 | 47.0 | 2.0 | 0.5 | Normal | 179.444 |
| T0328 | 90.0 | 4.0 | 1.8 | Normal | 192.935 |
| T0168 | 48.0 | 1.5 | 0.4 | Normal | 201.716 |
| T0303 | 21.0 | 1.8 | 1.3 | Normal | 211.310 |
| T0214 | 65.0 | 2.4 | 1.1 | Normal | 212.542 |
| T0094 | 77.0 | 4.7 | 4.0 | Normal | 213.166 |
| T0173 | 48.0 | 1.7 | 0.6 | Normal | 215.401 |
| T0297 | 69.0 | 1.3 | 1.0 | Normal | 217.279 |
| T0253 | 80.0 | 1.0 | 0.7 | Normal | 220.222 |
| T0229 | 27.0 | 1.6 | 0.5 | Normal | 221.885 |
| T0257 | 81.0 | 3.1 | 0.8 | Normal | 226.157 |
| T0109 | 66.0 | 1.9 | 0.7 | Normal | 226.895 |
| T0325 | 91.0 | 2.3 | 1.2 | Normal | 253.924 |
| T0286 | 68.0 | 2.8 | 1.3 | Normal | 255.798 |
| T0258 | 84.0 | 4.0 | 0.7 | Normal | 257.093 |
| T0343 | 68.0 | 2.1 | 0.5 | Normal | 267.476 |
| T0179 | 75.0 | 1.4 | 0.8 | Normal | 272.348 |
| T0135 | 83.0 | 3.4 | 0.8 | Normal | 285.744 |
| T0207 | 66.0 | 0.8 | 0.6 | Normal | 286.857 |
| T0350 | 22.0 | 3.3 | 1.4 | Normal | 293.384 |
| T0108 | 83.0 | 1.1 | 0.9 | Normal | 296.460 |
| T0331 | 63.0 | 3.7 | 2.3 | Normal | 309.276 |
| T0311 | 28.0 | 12.3 | 10.9 | Normal | 312.038 |
| T0357 | 72.0 | 1.6 | 0.7 | Normal | 312.487 |
| T0138 | 83.0 | 1.3 | 0.8 | Normal | 319.250 |
| T0095 | 83.0 | 1.8 | 1.7 | Normal | 336.488 |
| T0153 | 88.0 | 1.8 | 1.3 | Normal | 346.562 |
| T0205 | 72.0 | 4.3 | 2.4 | Normal | 358.092 |
| T0196 | 94.0 | 2.3 | 1.5 | Normal | 368.722 |
| T0137 | 85.0 | 18.0 | 17.0 | Normal | 374.100 |
| T0281 | 75.0 | 3.6 | 3.1 | Normal | 386.356 |
| T0152 | 90.0 | 1.8 | 0.8 | Normal | 412.211 |
| T0132 | 70.0 | 1.4 | 0.5 | Normal | 414.939 |
| T0161 | 82.0 | 8.8 | 7.9 | Normal | 417.934 |
| T0263 | 89.0 | 1.5 | 1.0 | Normal | 426.157 |
| T0193 | 37.0 | 1.1 | 0.5 | Normal | 430.407 |
| T0225 | 73.0 | 9.1 | 7.1 | Normal | 434.398 |
| T0182 | 24.0 | 2.0 | 0.5 | Normal | 439.319 |
| T0172 | 77.0 | 5.7 | 1.1 | Normal | 441.014 |
| T0102 | 75.0 | 14.8 | 9.5 | Normal | 456.852 |
| T0212 | 78.0 | 5.2 | 0.7 | Normal | 480.616 |
| T0219 | 58.0 | 4.3 | 0.7 | Normal | 513.266 |
| T0177 | 94.0 | 2.7 | 1.4 | Normal | 517.067 |
| T0278 | 86.0 | 9.0 | 8.3 | Normal | 526.452 |
| T0273 | 93.0 | 1.9 | 1.1 | Normal | 529.582 |
| T0139 | 76.0 | 2.6 | 1.7 | Normal | 535.240 |
| T0176 | 22.0 | 3.1 | 1.1 | Normal | 547.874 |
| T0231 | 84.0 | 3.3 | 1.6 | Normal | 559.083 |
| T0184 | 64.0 | 8.4 | 2.5 | Normal | 582.397 |
| T0192 | 93.0 | 5.3 | 1.9 | Normal | 583.007 |
| T0163 | 77.0 | 1.7 | 0.5 | Normal | 593.990 |
| T0159 | 78.0 | 1.4 | 1.1 | Normal | 596.170 |
| T0104 | 100.0 | 4.9 | 0.9 | Normal | 603.806 |
| T0103 | 73.0 | 17.0 | 15.1 | Normal | 614.455 |
| T0140 | 85.0 | 4.1 | 0.7 | Normal | 632.945 |
| T0310 | 81.0 | 11.7 | 11.2 | Normal | 637.862 |
| T0174 | 92.0 | 15.9 | 14.3 | Normal | 784.946 |
| T0353 | 61.0 | 3.5 | 2.1 | Normal | 795.052 |
| T0187 | 74.0 | 1.2 | 0.7 | Normal | 817.190 |
| T0206 | 76.0 | 1.2 | 0.9 | Normal | 841.504 |
| T0142 | 88.0 | 2.9 | 2.5 | Normal | 873.413 |
| T0134 | 18.0 | 2.3 | 0.5 | Normal | 901.565 |
| T0283 | 88.0 | 5.2 | 2.0 | Normal | 903.330 |
| T0124 | 92.0 | 18.8 | 13.4 | Normal | 987.762 |
| T0272 | 29.0 | 4.2 | 1.2 | Normal | 1025.266 |
| T0237 | 88.0 | 4.7 | 1.4 | Normal | 1133.390 |
| T0268 | 69.0 | 3.1 | 0.9 | Normal | 1283.277 |
| T0199 | 81.0 | 3.8 | 1.7 | Normal | 1288.335 |
| T0306 | 41.0 | 3.9 | 0.7 | Normal | 1353.578 |
| T0211 | 85.0 | 16.1 | 14.6 | Normal | 1614.338 |
| T0245 | 78.0 | 5.4 | 1.1 | Normal | 1651.881 |
| T0364 | 19.0 | 4.9 | 4.1 | Normal | 1990.671 |
| T0315 | 89.0 | 3.6 | 2.2 | Normal | 4027.681 |
| T0190 | 47.0 | 2.8 | 1.7 | Normal | 8271.513 |
| T0002 | 67.0 | 4.9 | 2.7 | Normal | N/A |
| T0005 | 84.0 | 3.2 | 1.3 | Normal | N/A |
| T0007 | 41.0 | 3.2 | 0.6 | Normal | N/A |
| T0011 | 85.0 | 2.6 | 2.4 | Normal | N/A |
| T0013 | 75.0 | 2.1 | 1.6 | Normal | N/A |
| T0014 | 70.0 | 5.2 | 2.0 | Normal | N/A |
| T0016 | 66.0 | 6.4 | 3.9 | Normal | N/A |
| T0017 | 73.0 | 17.8 | 13.1 | Normal | N/A |
| T0018 | 20.0 | 0.4 | 0.2 | Normal | N/A |
| T0019 | 88.0 | 2.4 | 0.8 | Normal | N/A |
| T0020 | 83.0 | 2.5 | 1.1 | Normal | N/A |
| T0022 | 34.0 | 1.3 | 0.3 | Normal | N/A |
| T0023 | 59.0 | 1.5 | 0.7 | Normal | N/A |
| T0024 | 89.0 | 1.8 | 0.7 | Normal | N/A |
| T0030 | 59.0 | 23.7 | 23.4 | Normal | N/A |
| T0031 | 82.0 | 1.3 | 1.0 | Normal | N/A |
| T0032 | 32.0 | 1.0 | 0.5 | Normal | N/A |
| T0033 | 30.0 | 1.7 | 1.0 | Normal | N/A |
| T0034 | 36.0 | 1.2 | 0.7 | Normal | N/A |
| T0037 | 74.0 | 1.4 | 1.2 | Normal | N/A |
| T0038 | 77.0 | 2.0 | 1.1 | Normal | N/A |
| T0042 | 88.0 | 2.4 | 2.1 | Normal | N/A |
| T0045 | 80.0 | 7.1 | 2.7 | Normal | N/A |
| T0048 | 26.0 | 22.8 | 22.2 | Normal | N/A |
| T0051 | 68.0 | 2.3 | 1.2 | Normal | N/A |
| T0052 | 72.0 | 5.5 | 3.0 | Normal | N/A |
| T0053 | 86.0 | 9.8 | 8.3 | Normal | N/A |
| T0057 | 57.0 | 6.3 | 3.2 | Normal | N/A |
| T0058 | 85.0 | 17.8 | 9.1 | Normal | N/A |
| T0059 | 90.0 | 2.1 | 1.4 | Normal | N/A |
| T0060 | 83.0 | 1.7 | 0.4 | Normal | N/A |
| T0063 | 49.0 | 1.0 | 0.7 | Normal | N/A |
| T0068 | 87.0 | 6.3 | 6.1 | Normal | N/A |
| T0070 | 75.0 | 1.1 | 0.6 | Normal | N/A |
| T0071 | 64.0 | 13.2 | 12.4 | Normal | N/A |
| T0076 | 88.0 | 14.9 | 13.4 | Normal | N/A |
| T0077 | 77.0 | 1.9 | 0.6 | Normal | N/A |
| T0078 | 35.0 | 11.1 | 6.7 | Normal | N/A |
| T0079 | 71.0 | 1.3 | 0.9 | Normal | N/A |
| T0080 | 85.0 | 2.8 | 1.0 | Normal | N/A |
| T0081 | 62.0 | 1.7 | 0.8 | Normal | N/A |
| T0085 | 86.0 | 3.8 | 2.9 | Normal | N/A |
| T0100 | 61.0 | 3.9 | 3.4 | Normal | N/A |
| T0101 | 21.0 | 2.3 | 0.9 | Normal | N/A |
| T0210 | 56.0 | N/A | 1.1 | Normal | N/A |
| T0244 | 81.0 | 16.3 | 15.0 | Normal | N/A |
| T0308 | 85.0 | 3.2 | 1.0 | Normal | N/A |
| T0320 | 68.0 | 5.7 | 5.0 | Normal | N/A |
| T0322 | 92.0 | 3.8 | 1.3 | Normal | N/A |
| T0323 | 53.0 | 5.9 | 4.9 | Normal | N/A |
| T0326 | 75.0 | N/A | 1.0 | Normal | N/A |
| T0348 | 66.0 | 3.5 | 0.8 | Normal | N/A |
| T0356 | 52.0 | 3.7 | 0.9 | Normal | N/A |
| T0271 | 73.0 | 4.6 | 3.4 | Abnormal | 139.631 |
| T0355 | 96.0 | 1.3 | 1.1 | Abnormal | 235.882 |
| T0267 | 89.0 | 2.9 | 2.2 | Abnormal | 243.490 |
| T0295 | 86.0 | 2.9 | 1.6 | Abnormal | 315.244 |
| T0277 | 73.0 | 1.3 | 0.3 | Abnormal | 332.700 |
| T0113 | 65.0 | 1.4 | 1.2 | Abnormal | 343.830 |
| T0351 | 72.0 | 4.3 | 2.3 | Abnormal | 437.059 |
| T0120 | 76.0 | 1.3 | 0.9 | Abnormal | 445.637 |
| T0107 | 71.0 | 8.7 | 8.2 | Abnormal | 472.407 |
| T0228 | 87.0 | 2.5 | 1.7 | Abnormal | 541.537 |
| T0360 | 92.0 | 1.4 | 0.4 | Abnormal | 630.180 |
| T0230 | 88.0 | 4.5 | 1.4 | Abnormal | 886.358 |
| T0175 | 85.0 | 2.6 | 1.2 | Abnormal | 970.906 |
| T0198 | 80.0 | 1.3 | 1.0 | Abnormal | 1001.789 |
| T0189 | 61.0 | 5.1 | 1.2 | Abnormal | 1490.067 |
| T0232 | 77.0 | 2.9 | 1.5 | Abnormal | 1792.664 |
| T0157 | 89.0 | 4.8 | 2.6 | Abnormal | 1862.961 |
| T0235 | 83.0 | 5.3 | 1.2 | Abnormal | 1941.208 |
| T0321 | 82.0 | 3.9 | 3.2 | Abnormal | 2318.664 |
| T0302 | 68.0 | 3.3 | 2.3 | Abnormal | 2857.223 |
| T0338 | 87.0 | 7.1 | 4.5 | Abnormal | 2859.929 |
| T0246 | 95.0 | 5.9 | 2.5 | Abnormal | 3152.792 |
| T0200 | 42.0 | 2.1 | 1.3 | Abnormal | 3768.306 |
| T0215 | 72.0 | 3.8 | 3.4 | Abnormal | 4296.402 |
| T0363 | 89.0 | 8.3 | 5.9 | Abnormal | 6838.576 |
| T0012 | 54.0 | 3.3 | 2.0 | Abnormal | N/A |
| T0029 | 85.0 | 5.9 | 5.7 | Abnormal | N/A |
| T0040 | 68.0 | 8.8 | 2.0 | Abnormal | N/A |
| T0047 | 75.0 | 19.4 | 18.9 | Abnormal | N/A |
| T0105 | 79.0 | 2.4 | 0.7 | Abnormal | N/A |
| T0155 | 51.0 | 1.0 | 0.6 | Abnormal | N/A |
